# Management Using V-Y Plasty Approach for Abnormal Frenal Attachment: A Case Report

**DOI:** 10.7759/cureus.57663

**Published:** 2024-04-05

**Authors:** Unnati Shirbhate, Pavan Bajaj, Ranu Oza, Dhruvi Solanki

**Affiliations:** 1 Department of Periodontics, Sharad Pawar Dental College, Datta Meghe Institute of Higher Education and Research, Wardha, IND; 2 Department of Pedodontics and Preventive Dentistry, Sharad Pawar Dental College, Datta Meghe Institute of Higher Education and Research, Wardha, IND

**Keywords:** surgical excision, laser, v-y plasty, frenectomy, periodontal health, aberrant frenum

## Abstract

The mucous membrane fold, which facilitates the attachment of the gingiva, alveolar mucosa, and the periosteum surrounding the lips and cheek, is known as the frenum. The frenal attachment at the gingival or papillary level may comprise periodontal health due to difficulty with plaque adherence or muscle pull. The management of such aberrant frenal attachment becomes necessary to avoid the associated future problems, such as midline diastema and periodontal attachment loss, which might lead to aesthetic problems and tooth mobility. The treatment modalities involve frenectomy using Miller's technique, conventional technique, Z-plasty, and V-Y plasty types of frenectomy procedures. The patient's requirements, specific indications, and intended results determine the method. This case report illustrates the utilisation of the V-Y plasty technique for the frenectomy of a papillary-type labial frenal attachment in a 19-year-old female patient. V-Y plasty proved to be an efficient technique for removing the aberrant labial frenum attachment, and the results were highly satisfactory, with less scar formation. V-Y plasty is reliable for covering defects and elongating the frenum area, giving desired clinical outcomes.

## Introduction

The fold of mucous membrane that connects the upper lip to the gingiva, alveolar mucosa, and periosteum is known as the maxillary labial frenum or frenulum labii superioris [1]. Histologically, it comprises mucous glands in the subcutaneous tissue, abundant elastic fibres, and loose connective tissue fibres [2]. A normal frenal attachment is typically present at the terminal part of the mucogingival junction to avoid the excessive pulling of marginal and attached gingiva. However, its level is variable, ranging from the vestibular depth, the crest of the alveolar ridge, and sometimes even that of the incisal papillary region of the anterior maxilla [3,4]. The ectolabial bands that join the palatine papilla to the tubercle of the upper lip give rise to the maxillary labial frenum as a post-eruptive remnant. With the eruption of the central incisors, no bone is produced beneath the frenum. Between the central incisors, there is a V-shaped bone cleft that leads to an aberrant frenal attachment [5].

Based on attachment and morphology, the literature has described several classifications of maxillary labial frenal attachments. Mirko et al. classified frenum in 1974 based on the extent of attachment of muscle fibres. (1) mucosal: attachment of frenal fibres up to the mucogingival junction, (2) gingival: frenal fibres inserted within attached gingiva, (3) papillary: extension of frenal fibres into interdental papilla, and (4) papilla penetrating: frenal fibres extend up to palatine papilla by crossing the alveolar process [6].

Treatment options for the aberrant frenum include frenectomy and frenotomy procedures. A frenectomy obliterates the frenum, including its attachment to the underlying bone, whereas a frenotomy entails making an incision and shifting the frenal attachment. Aberrant frenum has been corrected surgically using various surgical procedures, such as conventional technique using a scalpel, Miller's approach, V-Y plasty, Z-plasty, and paralleling technique [7]. One of the methods is the V-Y plasty technique, which is indicated for elongating the area with an abnormal frenal attachment to improve esthetic appearance in midline diastema cases, and also less scar formation is maintained [8]. V-Y plasty is a prevalent technique used in plastic surgery procedures. In this procedure, a V-shaped incision is made on the lower part of the frenal attachment, followed by an undermining of peripheral tissues [7,8].

This case presentation demonstrates the V-Y plasty procedure using a scalpel for aberrant frenal attachment after orthodontic closure of the midline diastema.

## Case presentation

**Figure 1:**
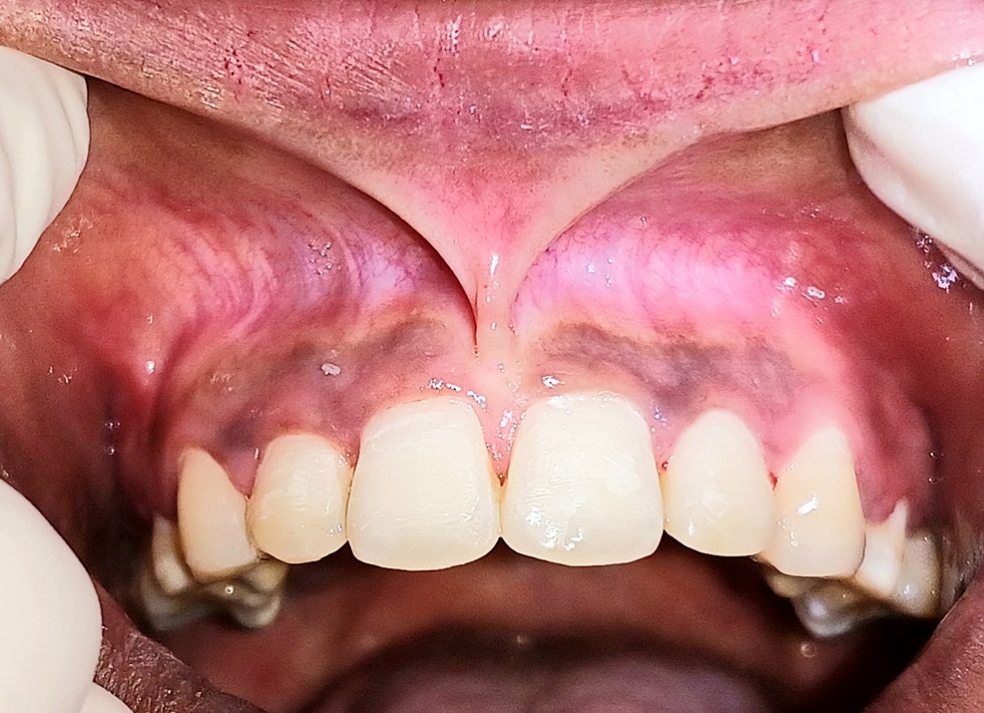
Pre-operative view of papillary type of frenal attachment after orthodontic midline closure

A 19-year-old female patient was referred from the Department of Orthodontics to the Department of Periodontics following the completion of orthodontic therapy to manage the papillary type of maxillary frenal attachment (Figure 1). The patient initially complained of midline diastema before orthodontic closure and was advised of a frenectomy procedure after diastema closure.

**Figure 2:**
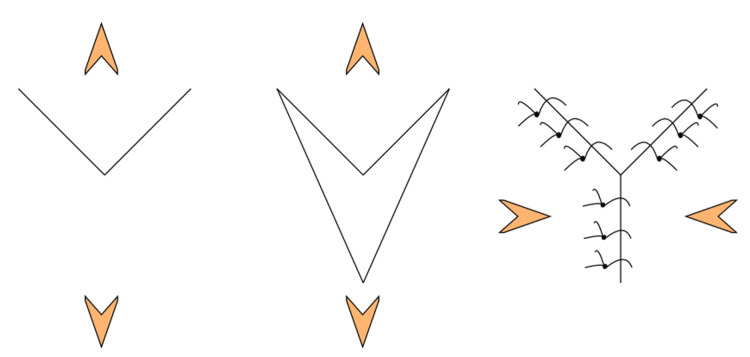
Procedural steps of incisions and suturing in V-Y plasty procedure

Labial frenectomy decisions can be made before or during the procedure, just before or after the diastema, by using orthodontic treatment in patients of high frenal attachment associated with midline diastema, depending upon the specifications of the cases. A thorough medical history was recorded. The patient gave informed written consent after being informed of the necessary surgical treatment. Following a routine haematological examination, the findings were within the expected range. Oral prophylaxis was done before the surgical removal of the frenum. In the V-Y plasty procedure, the apical point of the frenum is then moved, and the V-shaped incision is changed to Y shape with the help of appropriate suturing (Figure 2).

**Figure 3:**
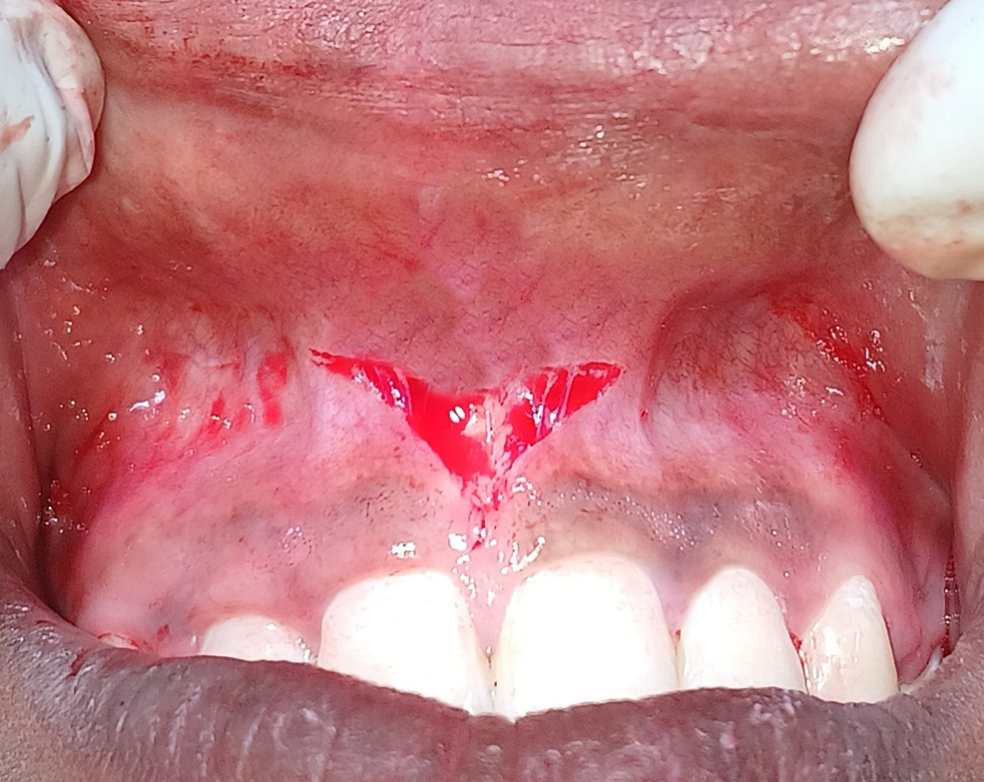
Frenectomy procedure performed with V-Y plasty using conventional scalpel technique

Frenectomy was carried out under aseptic conditions, precautions, and under local anaesthesia. The frenum was held using the hemostat, and the V-shaped incision was made on the lower part of the frenal attachment. Appropriate undermining of the peripheral tissues was done to help with proper flap placement and minimise the stretching of the underlying structures (Figure 3).

**Figure 4:**
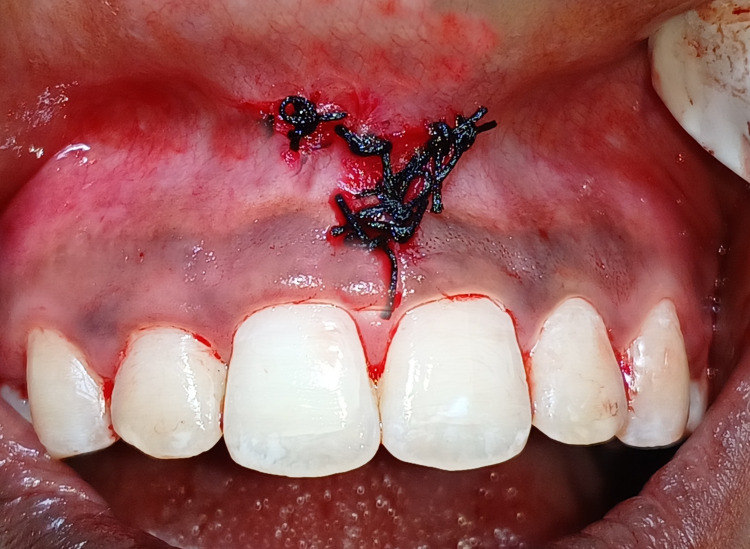
Sutures placed by maintaining V-Y shape of the incision

Simple interrupted suturing was done at the incision line using silk sutures (Figure 4). This maintained the V-Y shape at the surgical site. The patient was instructed about postoperative instructions for suture removal after seven days.

**Figure 5:**
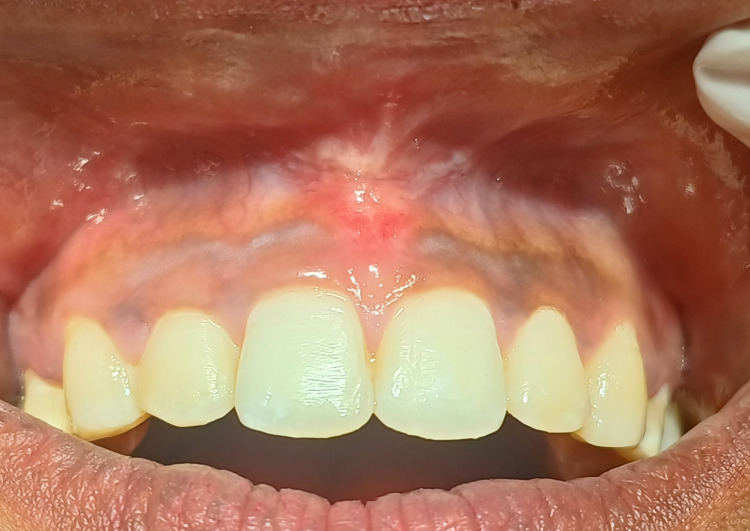
Post-operative view after seven days of the surgical site showing satisfactory healing

For suture removal, the patient revisited after seven days, showing satisfactory healing without any infective signs of swelling or infection and no pain or discomfort (Figure 5).

**Figure 6:**
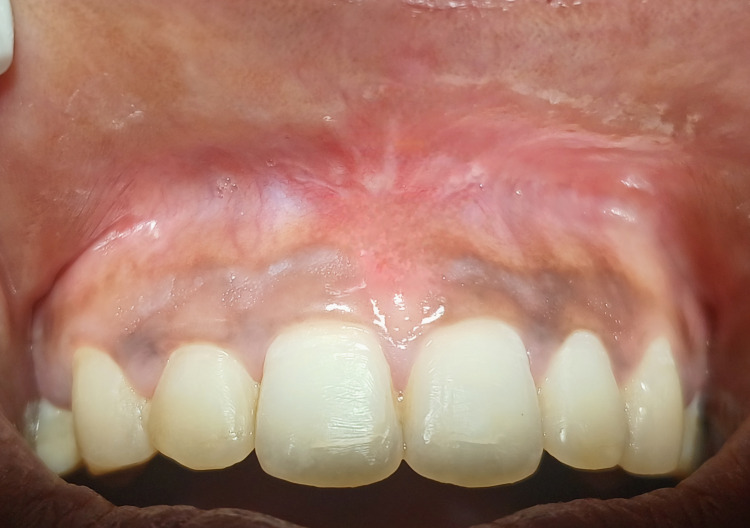
Patient reviewed after three months showing complete satisfactory healing and no scar formation of the surgical site

The patient was reviewed after three months and showed complete satisfactory healing, no recurrence sign, no scar formation, and no discomfort, maintaining aesthetic at the surgical site (Figure 6).

## Discussion

Aberrant maxillary frenal attachment leads to various periodontal complications, such as increased plaque accumulation, midline diastema, gingival recession, and compromised oral hygiene that may lead to periodontitis, unaesthetic appearance, and sometimes tooth loss [9]. A high frenum attachment often causes persistent diastemas during or after the orthodontic closure. Using a scalpel, the frenum is excised using the conventional technique. Frenectomy has also been proposed using lasers and electrosurgery [10]. The traditional approach of performing frenectomy procedures with a scalpel is comparatively simple but technique-sensitive. It promotes better tissue handling and healing but has the drawback of a longitudinal surgical incision that can result in periodontal problems and an unaesthetic appearance. One of the significant drawbacks is scar formation, which prevents the closure of midline diastema during orthodontic treatment. Due to this, frenectomy before orthodontic treatment is contraindicated in the case of midline diastema [11].

Several surgical technique modifications, including Miller's technique, V-Y plasty, and Z-plasty, have been performed since the conventional procedure of frenectomy was first offered to address the issues arising from an aberrant labial frenum [12]. Bajaj et al. used the Z-plasty technique to achieve satisfactory and desirable outcomes in abnormal frenal attachment with less scar tissue formation [13]. Phadnaik et al., in their case series, treated five cases of a classical frenectomy, V-Y plasty technique, Miller’s technique, Z-plasty, and frenectomy using a diode laser. They have concluded that according to the type of aberrant frenal attachments, all the above methods can be employed based on the indicated situations. Thus, choosing the correct procedure is the first step towards achieving outstanding functional and aesthetic results [14]. Kundu et al., in their case report, treated aberrant frenal attachment by employing the V-Y plasty technique using a scalpel where at one month of follow-up, it became apparent that the frenal attachment had been moved to an apical position, where scar tissue formation was minimum, and the healing was uneventful. The V-Y plasty technique shows lesser scar tissue formation than the conventional technique; hence, it can always be employed in managing aberrant frenal attachment cases with less scar formation and giving an aesthetically pleasant appearance [15].

## Conclusions

V-Y plasty is a strategically minimal invasive approach to aberrant frenum management. It treats aberrant high maxillary frenal attachments with minimum scarring and gives aesthetic benefits to the patient. It allows for the easy manipulation of fibrous tissue essential for addressing aberrant frenal attachment problems. The papillary frenal attachment demonstrated satisfactory results with proper postoperative healing and satisfactory results to the patient. It is a reliable and helpful method for covering the defects caused by frenectomy and elongating the structures with better clinical outcomes. This technique is reliable as it is relatively simple, does not involve bulk tissue removal like conventional technique, and is easy to perform, requiring less time and less surgical complexity.
